# Cutaneous Squamous Cell Carcinoma and Lichen Simplex Chronicus Successfully Treated with Topical Cannabinoid Oil: A Case Report and Summary of Cannabinoids in Dermatology

**DOI:** 10.7759/cureus.23850

**Published:** 2022-04-05

**Authors:** Jennifer Laborada, Philip R Cohen

**Affiliations:** 1 Medicine, University of California Riverside School of Medicine, Riverside, USA; 2 Dermatology, University of California Davis Medical Center, Sacramento, USA; 3 Dermatology, Touro University California College of Osteopathic Medicine, Vallejo, USA

**Keywords:** squamous cell carcinoma, skin, side effect, marijuana, lichen simplex chronicus, dermatology, cutaneous, cannabinoids, adverse event, psychoactive drug

## Abstract

Cannabidiol is a member of the cannabinoids, consisting of a diverse class of compounds derived from *Cannabis sativa*. There are three types of cannabinoids based on origin: endocannabinoids (endogenous), phytocannabinoids (plant-derived), and synthetic cannabinoids (synthesized). The endocannabinoid system plays a key role in skin homeostasis, such as proliferation, differentiation, and inflammatory signaling. A 64-year-old woman with a history of multiple squamous cell carcinomas who presented with skin lesions on her bilateral dorsal hands is reported. Her skin biopsies showed lichen simplex chronicus on her left hand and squamous cell carcinoma on her right hand; both lesions resolved with topical application of 20% cannabidiol. Cutaneous adverse events associated with cannabinoid use and potential therapeutic uses of cannabinoids in inflammatory skin diseases and skin cancer are also summarized.

## Introduction

Cannabinoids are a broad class of chemical compounds that are biologically and structurally similar to delta (9)-tetrahydrocannabinol, a major psychoactive compound derived from the plant *Cannabis sativa*, also known as marijuana. Cannabinoids are classified into three classes, depending on their origin: endocannabinoids, phytocannabinoids, and synthetic cannabinoids. Endocannabinoids are produced in animals and humans and present in the central and peripheral organs. Phytocannabinoids are produced from *C. sativa*, the most well-known being delta (9)-tetrahydrocannabinol and cannabidiol. Synthetic cannabinoids have been created in the laboratory [1].

Cannabinoids find several potential applications in dermatology. These include not only cosmetic uses, but also the management of several cutaneous diseases. In addition, possibly secondary to anti-inflammatory effects, cannabinoids may have a potential role in the management of cutaneous malignancy [2].

A woman with multiple biopsy-confirmed cutaneous squamous cell carcinomas presented with a new red plaque on her dorsal left hand. Biopsy revealed lichen simplex chronicus; however, she self-initiated treatment with topical cannabinoid oil, twice daily, and the lesion completely resolved within four weeks. Several prior biopsy-confirmed squamous cell carcinomas on her dorsal hands also completely regressed after similar treatment with topical cannabinoid oil. The potential role of cannabinoids in dermatology and observed adverse cutaneous events to topical and oral cannabinoids are summarized.

## Case presentation

A 64-year-old woman presented for an evaluation of her skin. She had a history of multiple squamous cell carcinomas predominantly restricted to her extensor forearms and dorsal hands. Many of these had been biopsy-confirmed and treated with a topical cannabinoid.

**Figure 1:**
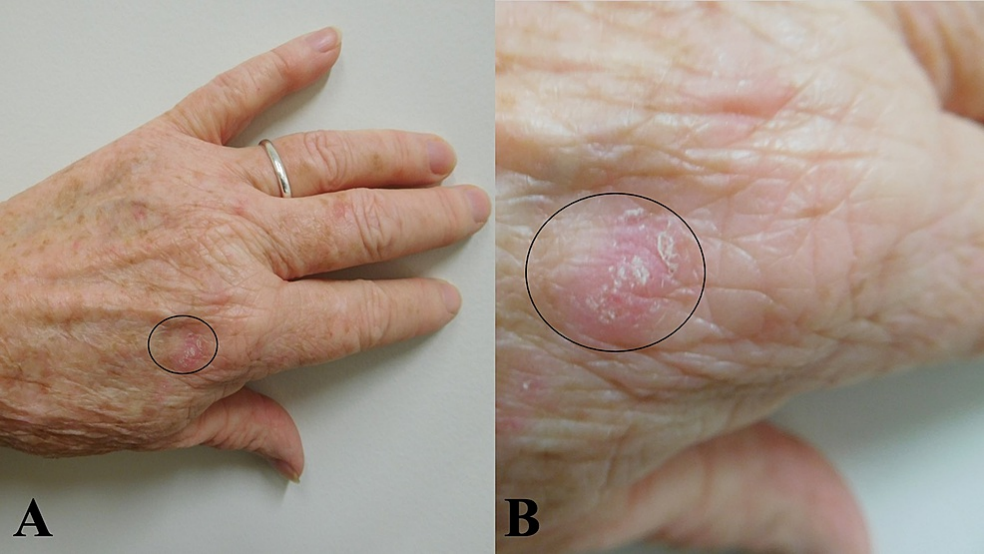
Lichen simplex chronicus on the left dorsal hand Distant (a) and closer (b) views of a red scaly plaque (black oval) on the left dorsal hand proximal to the metacarpophalangeal joint of the second digit. Skin biopsy established a diagnosis of lichen simplex chronicus, which completely resolved within four weeks after starting twice-daily topical treatment with 20% cannabinoid oil.

Cutaneous examination showed a new erythematous scaly 14 x 10 mm plaque on her left dorsal hand (Figure 1). The lesion had been present for three months. A skin biopsy demonstrated lichen simplex chronicus.

**Figure 2:**
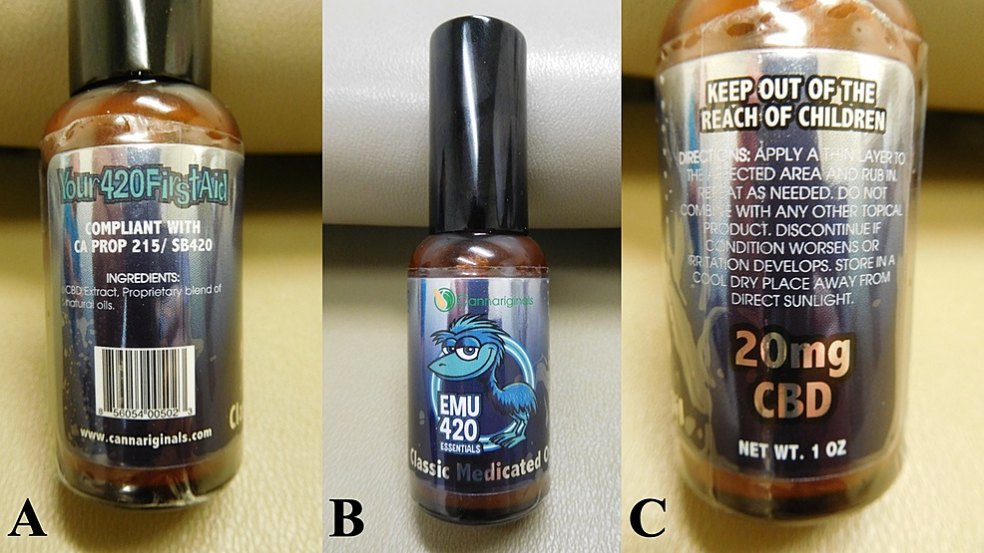
Cannabidiol oil components The list of ingredients (a) for a cannabinoid product containing 20 mg of cannabidiol in emu oil (EMU 420 essential) (b). The directions for topical application are also provided (c). CBD, cannabidiol.

Before receiving the pathology report, she began topical treatment of the lesion with a cannabinoid oil she had acquired that consisted of 20 mg of cannabidiol in emu oil (EMU 420 essential) (Figure 2). She applied the cannabinoid oil twice daily to the lesion. Within four weeks, the lesion site only showed erythema.

**Figure 3:**
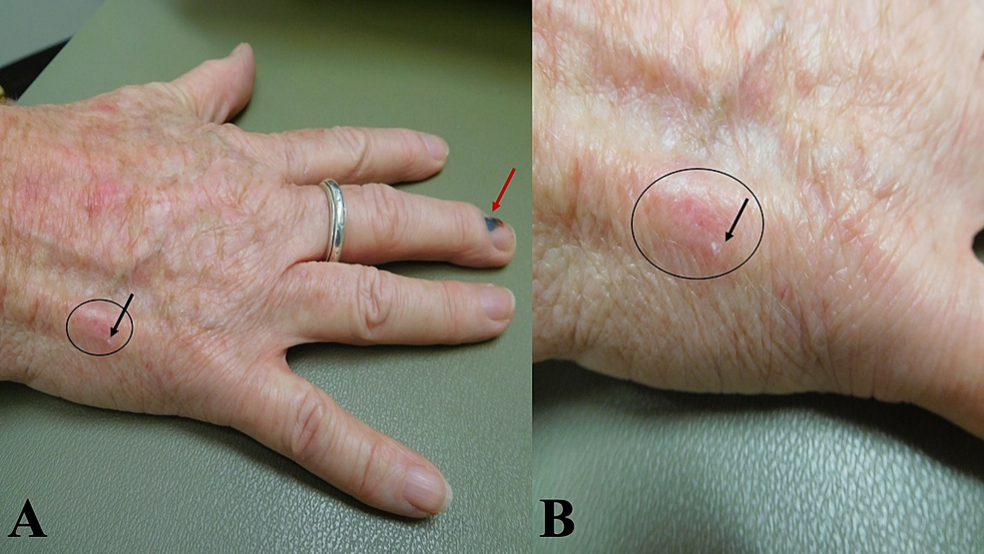
Resolved squamous cell carcinoma on the right hand Distant (a) and closer (b) views of an erythematous patch (black oval) with a small focus of scale (black arrow) on the lateral right dorsal hand representing the treated site of a biopsy-confirmed squamous cell carcinoma that completely resolved with twice daily application of CBD oil for four weeks.  A subungual hemorrhage is also present on the right middle finger (red arrow). CBD, cannabidiol.

Her lateral right hand showed the site of biopsy-confirmed squamous cell carcinoma. The original lesion had presented as an erythematous scaling red plaque. Currently, there was only a small focus of scale with an underlying 12 x 9 mm of erythema (Figure 3). She had treated the tumor with the same cannabinoid oil, twice daily; within less than four weeks, the tumor had completely resolved.

She commented that similar to the tumor on her right hand, several other biopsy-confirmed squamous cell carcinomas on her dorsal hands had rapidly disappeared after treatment with the cannabinoid oil.

A lymph node examination was performed, and there was no palpable adenopathy in the neck, submental area, or axillae. She intends to maintain regularly scheduled follow-up appointments for skin and lymph node examination to ensure the persistence of tumor regression.

## Discussion

Cannabinoids are classified based on their origin. Endocannabinoids are produced in animals and humans (Table 1) [1-3]. Phytocannabinoids are produced by the plant *C. sativa* (Table 2) [1-3], and synthetic cannabinoids are synthesized (Table 3) [1-3]. These three types of cannabinoids elicit their effects by interacting with the endocannabinoid system, which comprises G-protein-coupled receptors cannabinoid receptor 1 (CB1) and cannabinoid receptor 2 (CB2) in the central nervous system and peripheral organs, respectively (Table 4) [1-3]. 

**Table 1:** Endocannabinoid members ^a^These are cognate compounds and not true endocannabinoids, as they do not bind to cannabinoid receptors 1 and 2.

Endocannabinoid
2-Arachidonoylglycerol (2-AG)
Anandamide (AEA) or *N*-arachidonoylethanolamine
*N*-arachidonoyl dopamine
Docosatetraenyl ethanolamide (DEA)
Homo linoleoyl ethanolamide (HEA)
Noladin ether
Oleoylethanolamide (OEA)^a^
Palmitoylethanolamide (PEA)^a^
Virodhamine

**Table 2:** Phytocannabinoid members

Phytocannabinoid
Cannabidiol (CBD)
Cannabidiolic acid (CBDA)
Cannabigerol (CBG)
Cannabichromene (CBC)
Cannabinol (CBN)
Cannabidivarin (CBDV)
Beta-carophyllene
Delta (9)-tetrahydrocannabinol (THC)
Tetrahydrocannabinolic acid (THCA)
Delta (9)-tetrahydrocannabivarin (THCV)

**Table 3:** Synthetic cannabinoid members

Synthetic cannabinoid
Ajulemic acid (AJA)
CP 55, 940
Dronabinol
JWH-015
JWH-133
(R)-methanandamide (MET)
Nabilone
Nabiximols
WIN-55
212-2

**Table 4:** The endocannabinoid system CB, cannabinoid receptor; CNS, central nervous system.

Cannabinoid receptor	Location	Effects
CB1	CNS	Psychoactive
CB2	Immune cells, peripheral organs	Anti-inflammatory, immunomodulatory

There are several routes of administration for cannabinoids; indeed, the base likely affects the absorption level (Table 5) [4]. Topical application is most commonly used in dermatology. Cannabinoids may be used in smoking or used in baking brownies [4].

**Table 5:** Modes of cannabinoid delivery ^a^Available forms of topical application include balms, bath bombs, creams, lip balms, lotions, oils, ointments, salves, soaps, sticks/roll-ons, and transdermal delivery.

Cannabinoid delivery mode
Inhalation
Oral ingestion
Topical application^a^
Vaping

Cannabinoids may have therapeutic potential for the treatment of pruritus. In a prospective cohort study of 2456 patients with atopic dermatitis, application of a palmitoylethanolamide containing cream twice a day for four to six weeks was associated with a statistically significant improvement in itch and sleep disturbance. The investigators postulated that palmitoylethanolamide alleviates itch by inhibiting the activation of mast cells by binding to cannabinoid receptor 2 [5]. Indeed, the anti-pruritic effects of cannabinoids can also be utilized in several skin conditions such as atopic dermatitis, lichen simplex chronicus, prurigo nodularis, and uremic pruritus [2,6,7].

Cannabinoids have shown therapeutic potential for acne through their anti-inflammatory, anti-microbial, and anti-lipogenic activity in human sebocytes *in vitro* (Table 6) [3]. In addition, cannabinoids may have anti-aging effects and stimulate collagen synthesis and regulate basal cells in the epidermis [3]. They may also play a role in wound healing by promoting wound re-epithelization and scar formation [2].

**Table 6:** Cannabinoids for acne and skin rejuvenation CBD, cannabidiol.

Skin condition or restoration	Comments	Reference
Acne	CBD can exert anti-inflammatory, anti-microbial, and anti-lipogenic activity in human sebocytes *in vitro*. In a study of 11 Asian men, 3% cannabis seeds extract cream applied to the cheek twice daily for 12 weeks was associated with a statistically significant decrease in erythema and sebum compared to a base cream	[3]
Skin rejuvenation	Cannabinoids play a role in mediating the proliferation, differentiation, and survival of basal cells in the epidermis. Hemp seed hexane extracts can stimulate collagen synthesis *in vitro*	[3]

The endocannabinoid system has anti-inflammatory properties and hence is used in the management of chronic inflammatory skin diseases. In a study of 11 patients treated with 3% cannabis seeds cream, there was a significant decrease in erythema and sebum, compared to controls. The investigators hypothesize a possible therapeutic use of cannabinoids not only for acne, but also for seborrheic dermatitis [6].

Other studies have demonstrated that cannabinoids have immunomodulatory effects. These include antigen processing, inhibition of human keratinocyte proliferation, macrophage/T-cell interaction, and release of interleukin-2, nitric oxide, and tumor necrosis factor-alpha from immune cells. Thus, it has been postulated that cannabinoids may be a novel therapeutic option for psoriasis [7].

Cannabinoids may play a role in the treatment of epidermolysis bullosa by modulating the inflammatory response and levels of keratins. In an observational study of three children with epidermolysis bullosa, treatment with cannabidiol demonstrated significant improvement in pain, blistering, and wound healing [8]. Similar promising findings were observed in another study of three adults with epidermolysis bullosa who were treated with combined sublingual tetrahydrocannabinol and cannabidiol [2].

Synthetic cannabinoids also offer a therapeutic option for the management of skin conditions. A phase II clinical trial including 42 patients demonstrated that individuals with systemic sclerosis/scleroderma treated with ajulemic acid (a synthetic cannabinoid) had a statistically significant increase in the likelihood of improvement compared to controls. The researchers hypothesized that this occurred since the cannabinoids reduced extracellular matrix deposition and fibroblast proliferation rate [8]. Furthermore, since synthetic cannabinoids have been associated with hair loss by inducing the catagen phase of hair growth, cannabinoid antagonists may represent a potential treatment option for patients with hair loss disorders [5].

Cannabinoids have also been evaluated for their potential role in the management of skin cancer (Table 7) [2,9]. A study using the synthetic cannabinoid WIN-55,212-2 demonstrated the induction of apoptosis of Kaposi sarcoma endothelial cells and inhibition of angiogenesis. However, another study found that low-dose delta (9)-tetrahydrocannabinol facilitated the replication and transmission of the Kaposi sarcoma-associated herpesvirus [2].

**Table 7:** Cutaneous malignancies and cannabinoids CBD, cannabidiol.

Skin cancer	Comment	Ref
Kaposi sarcoma	Synthetic cannabinoid WIN-55,212-2 can induce apoptosis of Kaposi sarcoma-derived cell lines. CBD inhibits tumor cell growth and angiogenesis among Kaposi sarcoma-affected endothelial cells	[2]
Melanoma	Endocannabinoids, phytocannabinoids, and synthetic cannabinoids decrease non-melanoma skin cancer and melanoma growth *in vitro* and *in vivo* through cannabinoid receptor-dependent and independent pathways	[9]
Non-melanoma skin cancer	Mice deficient in cannabinoid receptors 1 and 2 had significantly lower rates of ultraviolet B-induced inflammation and skin carcinogenesis than wild-type mice	[9]

Similarly, there is contradicting evidence on the therapeutic potential of cannabinoids for non-melanoma skin cancer and melanoma. One group of investigators reported that endocannabinoids, synthetic cannabinoids, and phytocannabinoids decreased non-melanoma skin cancer and melanoma growth *in vitro* and *in vivo* through cannabinoid receptor-dependent and independent pathways. Yet, another study indicated that mice deficient in cannabinoid receptors 1 and 2 had significantly lower rates of ultraviolet B-induced inflammation and skin carcinogenesis than wild-type mice. The conflicting cannabinoid data observed in non-melanoma skin cancer and melanoma studies could result from various factors including that the effects of cannabinoids are dose-specific and that the *in vitro *studies may not have accounted for the tumor microenvironment [6,7,9].

Our patient has multiple squamous cell carcinomas and lichen simplex chronicus. She applied 20% cannabidiol in emu oil twice daily to her lesions. Both malignant cancer and chronic dermatitis completely resolved within four weeks of twice-daily treatment. We attribute the clearance of her skin tumor to the cannabidiol; however, there has been no study performed evaluating the treatment of cutaneous squamous cell carcinoma with topical emu oil.

Topical cannabinoids can be associated with cutaneous adverse events, such as allergic contact dermatitis and contact urticaria (Table 8) [10-16]. A study of dispensaries in Los Angeles, California, discovered that 84% of over-the-counter topical cannabinoids had one or more North American contact dermatitis group (NACDG) allergens [17]. In addition, with regard to molecular pathogenesis, *in vitro* studies have shown that cannabinoid receptor 2 not only modulates the maturation of dendritic cells, but also that haptenized dendritic cells have the potential to induce contact hypersensitivity responses in mice [13].

**Table 8:** Cannabinoids and adverse cutaneous events CBD, cannabidiol.

Adverse event	Comment	Reference
Acne, hair loss, and premature aging	Among 136 patients being treated for addiction to synthetic cannabinoids, the most common dermatologic findings were acne, grey hair, hair loss, hallowed cheeks, periorbital darkening, and premature aging	[10]
Acute generalized exanthematous pustulosis	Acute generalized exanthematous pustulosis is a type IV hypersensitivity reaction that manifests as an acute rash within 48 hours of being exposed to certain drugs, including antibiotics, antifungals, CBD, and cocaine	[11]
Allergic contact dermatitis	Several studies have demonstrated allergic contact dermatitis to cannabinoids. A study of 129 people showed 63 individuals to have a positive skin test for marijuana pollen. Another study found that 78 of 127 patients tested (61%) had a positive skin test for cannabis pollen. The specific mechanism of action has not been fully elucidated, but an *in vitro* study found that cannabinoid receptor 2 can mediate the ability of dendritic cells to induce hapten-induced contact hypersensitivity in mice, which is an established animal model for allergic contact dermatitis	[12,13]
Contact urticaria	A 29-year-old technician developed wheals, rhinitis, and headaches after two years of occupational exposure at Forensic Science Service. Patch testing yielded a positive result for cannabis leaf	[14]
Eczema herpeticum	Cannabinoid abuse may be an underestimated risk factor for triggering eczema herpeticum in patients with atopic dermatitis or Darier disease. Eczema herpeticum is caused by the reactivation of the latent herpes simplex virus	[15]
Erythema ab igne	Erythema ab igne is triggered by excessive heat exposure from heating devices, heating pads, or laptop use. It can also be seen as a complication of cannabis hyperemesis syndrome	[16]

Rare complications of chronic cannabis use have also been observed. These include erythema ab igne triggered by excessive heat among patients with concurrent cannabis hyperemesis syndrome. They also include cannabis arteritis, which presents as a severe peripheral vascular disease characterized by necrosis and ulceration of the limbs [16,18].

Oral cannabidiol and marijuana ingestion can, albeit uncommonly, cause acute generalized exanthematous pustulosis. This is a type IV hypersensitivity reaction that manifests as rapidly evolving pustules within 48 hours of the drug exposure [11]. In addition, anaphylaxis, a life-threatening complication, has been observed with ingestion of hempseed (*C. sativa*) [18].

Cannabis abuse can also have an immunosuppressive effect in patients with certain predisposing skin diseases and has been postulated to result in the reactivation of latent viruses such as the herpes simplex virus. A 22-year-old man with a history of cannabis abuse and Darier disease subsequently developed eczema herpeticum, which presented as a disseminated mucocutaneous eruption involving the oral mucosa, face, trunk, and groin. His viral infection gradually improved after receiving intravenous acyclovir (at a dose of 5 mg/kg three times a day) and parenteral nutrition [15].

Synthetic cannabinoid intoxication can present with psychiatric symptoms including agitation, anxiety, avoidant eye contact, delusions, paranoia, psychosis, and even suicidal ideation and violent behavior. Cannabis use has also been reported to exacerbate symptoms in individuals with schizophrenia. However, it is difficult to differentiate whether these symptoms are related to cannabis use or the underlying psychiatric illness [18,19].

## Conclusions

Cannabinoids are a class of drugs that are found in animals, humans, and plants; they are also synthesized. They are useful in the management of several systemic diseases. Indeed, cannabinoids have also been observed to be potentially effective in the treatment of cosmetic skin conditions and cutaneous diseases. In addition, they may be therapeutic in the management of not only non-melanoma skin cancer, such as squamous cell carcinoma, but also melanoma and Kaposi sarcoma. Our patient had successful treatment of a benign skin condition (lichen simplex chronicus); in addition, she had complete regression of several biopsy-confirmed squamous cell carcinomas. Therefore, the possibility of treating non-melanoma skin cancer, such as squamous cell carcinoma, with topical cannabinoids may warrant further investigation.
